# Beta-Catenin Haplo Insufficient Male Mice Do Not Lose Bone in Response to Hindlimb Unloading

**DOI:** 10.1371/journal.pone.0158381

**Published:** 2016-07-13

**Authors:** Delphine B. Maurel, Peipei Duan, Joshua Farr, An-Lin Cheng, Mark L. Johnson, Lynda F. Bonewald

**Affiliations:** 1 Department of Oral and Craniofacial Sciences, School of Dentistry, University of Missouri-Kansas City, Kansas City, MO, United States of America; 2 School of Nursing and Health Studies, University of Missouri-Kansas City, Kansas City, MO, United States of America; Université de Lyon - Université Jean Monnet, FRANCE

## Abstract

As the β-catenin pathway has been shown to be involved in mechanotransduction, we sought to determine if haploinsufficiency would affect skeletal response to unloading. It has previously been shown that deletion of both alleles of β-catenin in bone cells results in a fragile skeleton highly susceptible to fracture, but deletion of one allele using Dmp1-Cre (*Ctnnb1*^*+/loxP*^; *Dmp1-Cre*, cKO HET) has little effect on the 2 mo old skeleton. We found that under normal housing conditions, trabecular bone volume was significantly less in 5 mo old male cKO HET mice compared to controls (Ctrl/HET:Tb. BV/TV = 13.96±2.71/8.92±0.95%, Tb.N. = 4.88±0.51/3.95±0.44/mm, Tb. Sp. = 0.20±0.02/0.26±0.03mm, a 36%, 19% and 30% change respectively) but not in females suggesting an age and gender related effect. Before performing suspension experiments and to control for the environmental effects, animals with the same tail attachment and housing conditions, but not suspended (NS), were compared to normally housed (NH) animals. Attachment and housing resulted in weight loss in both genders and phenotypes. Cortical bone loss was observed in the cKO HET males (NH/NS, Ct BV/TV: 90.45±0.72/89.12±0.56%) and both diaphyseal (0.19±0.01/0.17±0.01mm) and metaphyseal (0.10±0.01/0.08±0.01mm) thickness, but not in female cKO HET mice suggesting that male cKO HET mice are susceptible to attachment and housing conditions. These results with transgenic mice emphasizes the importance of proper controls when attributing skeletal responses to unloading. With suspension, cKO HET male mice did not lose bone unlike female cKO HET mice that had greater trabecular bone loss than controls (Ctrl 9%:cKO HET 21% decrease Tb. N; Ctrl 12%:cKO HET 27% increase Tb. Sp.). Suspended and non-suspended mice lost weight compared to normally housed animals. Taken together, the data suggest a protective effect of β-catenin against the effects of stress in males and partial protection against unloading in females.

## Introduction

It has been recognized for hundreds of years that the skeleton responds to loading and unloading with bone gain or bone loss [1–3], but the cellular and molecular mechanisms responsible have remained elusive. The cells in bone that are thought to play a major role in sensing loading or unloading are osteocytes [4]. Osteocytes are ideally situated in bone to sense mechanical strain and translate that strain into signals for bone formation, bone resorption and regulation of mineralization [5]. These cells are embedded in the bone matrix throughout cortical and trabecular bone and are connected to each other, the bone marrow and the bone surface via their dendrites and canalicular system [6]. It has been shown that osteocytes are dynamic, active cells that can extend and retract their processes into marrow and vascular spaces [7]. Osteocytes are more sensitive to fluid flow shear stress than osteoblasts [8]. Primary osteocytes and the MLO-Y4 osteocyte-like cell line release nitric oxide, ATP and prostaglandins after being mechanically stimulated [8–10] and blocking these small molecular weight signaling molecules reduces or blocks anabolic responses of bone to loading [11]. Deletion of osteocytes results in bone loss and bone that does not respond to unloading [12]. In response to unloading, osteocytes have been shown to express greater RANKL/OPG ratios responsible for osteoclastogenesis [13]. These data show that osteocytes are mechanosensors and are required for maintaining bone mass. The role of osteocytes and their intracellular molecular mechanisms responsible for modulating strain effects on bone formation, resorption and mineralization are just beginning to be identified.

One pathway that has been explored as a modulator of the loading response in the osteocyte is the Wnt/β-catenin pathway. When the Wnt/ β-catenin pathway is activated, β-catenin is not degraded by the proteasome and will accumulate in the cytoplasm for translocation to the nucleus where it affects gene transcription. Activating receptors of this pathway include Lrp5 [14], Lrp6 [15], Lrp4 [16] and prostaglandin receptors [11]. Ligands include the Wnts and inhibitors include sclerostin [17] and Dkk1 [18]. Many of the components of this pathway have been shown to have important roles in regulating bone mass such as mutations in *LRP5* causing either high [19] or low [20] bone mass and the inhibition of osteoblastic bone formation by sclerostin, a protein highly expressed in osteocytes [21].

The Wnt/ β-catenin pathway is known to have an important role in the development of the skeleton, as well as maintenance of the adult skeleton [5]. The Wnt/β-catenin pathway regulates bone mass through various processes, including osteoblastogenesis, commitment of mesenchymal stem cells to the osteoblast lineage and osteoblastic precursor proliferation and differentiation [22–24]. Conditional deletion of the β-catenin gene in osteoblasts *in vivo* using alpha 1 type I collagen-Cre or osteocalcin-Cre revealed an essential function for β catenin-dependent Wnt signaling in controlling osteoclast differentiation and bone homeostasis. Osteoblast-specific β-catenin-deficient mice develop a low bone mass phenotype as a result of increased osteoclastic resorption due to decreased expression of osteoprotegerin [23–24]. Kramer and colleagues performed targeted deletion of β-catenin in osteocytes using the Dmp1-Cre promoter to assess the specific role of β-catenin in osteocytes. They showed that deletion of both alleles of β-catenin in osteocytes results in low bone mass and body weight, with an early lethality. Both cortical bone and trabecular bone was decreased in these animals [25]. A recent study by Javaheri et al. utilized the Dmp1-Cre β-catenin heterozygotes to determine if β-catenin plays a role in mechanotransduction [26]. While the control animals showed a significant response to anabolic load, the animals lacking one allele of β-catenin did not respond suggesting that β-catenin in osteocytes is essential for bone response to loading.

Unloading of bone has very significant effects on overall health. Lack of exercise [27], space flight [28] or long term bed rest [29] can have very detrimental effects on the skeleton. Understanding the cellular and molecular mechanisms responsible for bone loss due to unloading could potentially lead to prevention and new therapies. In this study, we hypothesized that mice with deletion of one allele of β-catenin in osteocytes (cKO HET) would have greater bone loss in response to unloading than control animals. Experiments were designed to answer three questions: 1). Is there a tibial bone phenotype in the cKO HET compared to controls? 2). Is there an effect of tail attachment and housing conditions? 3). What is the effect of unloading in cKO HET mice? The hypothesis was supported with regards to female cKO HET mice but not in male cKO HET mice. Male cKO HET mice lost bone with age and with the environmental stress of tail attachment and did not show additional bone loss with suspension. These studies show gender effects of β-catenin in bone.

## Materials and Methods

### Animals

The β-catenin fl/fl (exon 2–6) mice (controls) were purchased originally from Jackson Laboratories (B6.129-Catnbtm2Kem/J; Jackson Laboratories, Bar Harbor, ME, USA). The β-catenin fl/fl mice were crossed with the 10Kb Dmp1-Cre mice (30) to produce male and female mice with a heterozygous deletion of β-catenin in osteocytes (fl/+). All mice were genotyped in our laboratory. The primers sequences were the following: Dmp1-Cre (Forward primer: DGFPU: 5’-CCAAGCCCTGAAAATCACAGA-3’; Reverse primer: DGFPL: 5’-CCTGGCGATCCCTGAACATG-3’) and floxed β-Catenin (Forward primer: RM41: 5'-AAGGTAGAGTGATGAAAGTTGTT-3'; Reverse primer: RM42: 5'-CACCATGTCCTCTGTCTATTC-3'). Only the heterozygous β-catenin cKO HET mouse model (*Ctnnb1*^*+/loxP*^; *Dmp1-Cre*), were used as the homozygous mice (*Ctnnb1*^*loxP/loxP*^; *Dmp1-Cre*) do not generally survive past 3 months of age. The heterozygous mice survive under normal conditions and the only bone phenotype previously reported was 5% less trabecular bone in femurs in females at 2 months of age [25]. All mice were 14 ± 1 week old at baseline. All animal experiments were approved by the UMKC Institutional Animal Care and Use Committee.

### Tail suspension

**Fig 1:**
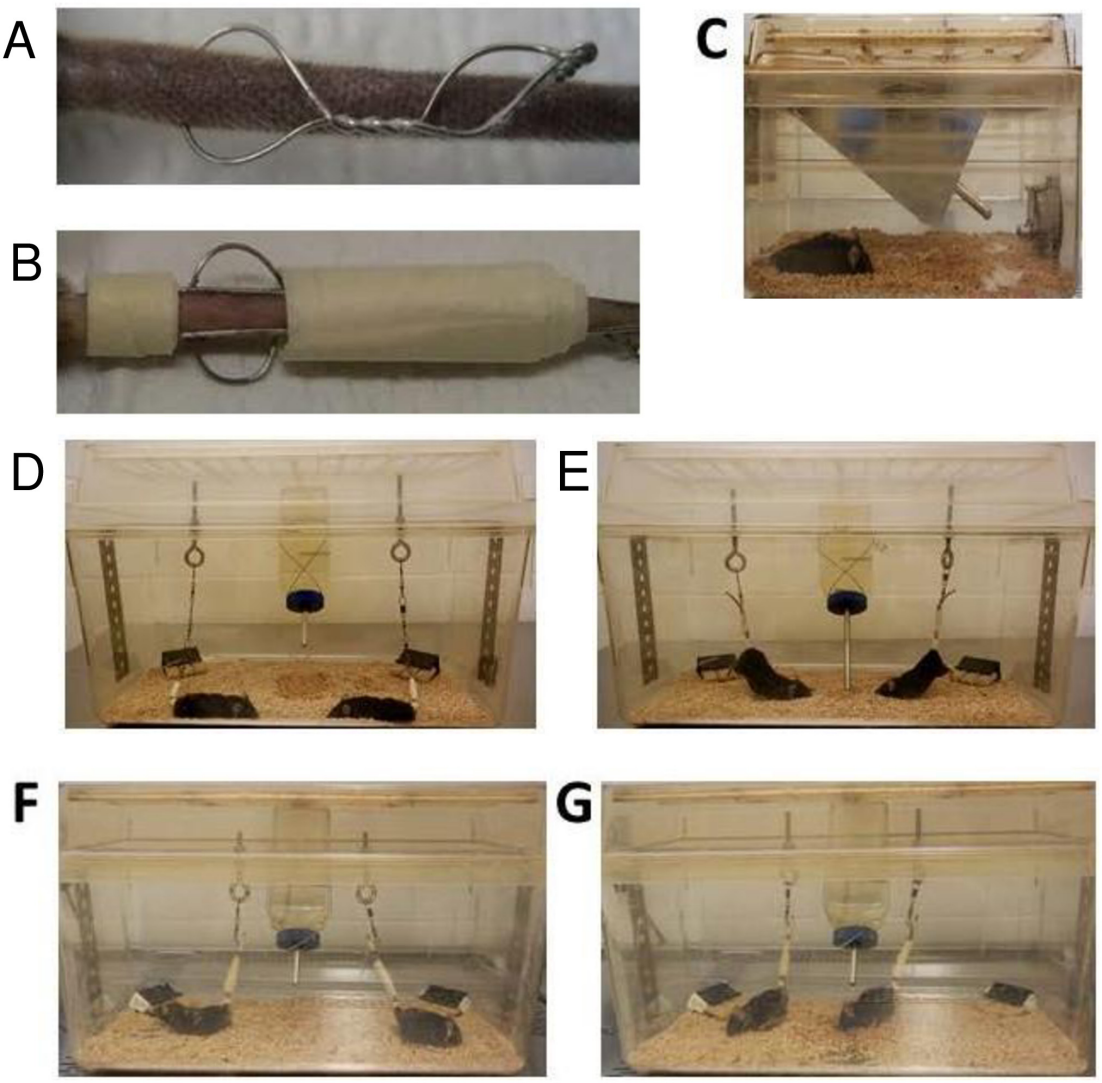
Housing Conditions. Illustration of the tail ring (A), and steel wires with tape (B) as applied to the tail for the suspension studies. Also shown are the three different housing conditions; normal housing (C), non suspended housing (D), and suspended housing (E) using terminated immobile mice to show angles of suspension as active mice in non suspended housing (F) and suspended (G) are difficult to photograph. The food blocks were clamped (black boxes) to allow access but to prevent movement of food block for resting of hindlimbs.

The tail suspension model has been used to study the effects of weightlessness as well as non-loading of bones [30]. We used a method adapted from Morey-Holton by Ferreira et al. [31] where a wire insertion between tail vertebrae which can occur with only taping. According to the authors, this method maintains weight with little stress, as the weight of the adrenal gland was not different between suspended compared to non-suspended animals. The advantage of this method is that the animals cannot escape (Fig 1).

Male and female β-catenin fl/+ mice (controls) and Dmp1 x β-catenin fl/+ (cKO HET) were used in these experiments. Three housing conditions were tested: normal housing (NH), non-suspended conditions (mice with a tail ring, attached but not suspended, NS) and suspended conditions (mice with a tail ring, attached and suspended, SUS). There were 5–8 animals per group, 82 animals in total. The experiment was repeated 7 times with different litters for each gender, genotype and condition. The animals were weighed at baseline and randomly assigned to one of the three housing groups.

Before surgical placement of the tail ring, at 14 weeks of age, the mice of the NS and SUS groups were anesthetized with 4% isoflurane inhalation (Isothesia, Isoflurane, USP, Butler Animal Health Supply, Dublin, OH, USA) using an isoflurane portable device (Isotec 4 vaporizer; Jorgensen Labs, Inc., Loveland, CO, USA). The tail was scrubbed with a sterile gauze, soaked in betadine, and rinsed with sterile water three times. Following the sterile procedure, a pilot hole was made with a 27G1/2 needle between two vertebrae into the intervertebral space, within 1 cm from the base of the tail. The needle was removed and replaced with a 10cm long piece of sterile surgical steel suture, wound and twisted to create a loop. (Fig 1). Bleeding was stopped with styptic powder if observed.

Initial experiments suggested that the tail ring was uncomfortable and was moving toward the surface of the skin due to uneven weight distribution. To distribute the weight, a surgical steel rod was placed before and after the tail ring and taped. This distributed the weight along several caudal vertebrae and was well tolerated by the mice. After insertion and anchoring of the tail ring, the mice were allowed to recover for 5–7 days and housed one per cage to prevent tail ring entanglement prior to hindlimb unloading. The cages were standard mice cages on a ventilated rack in a pathogen-free animal facility (Barrier type) where the temperature, humidity and light cycles are controlled (20–26°C, 30–70%, 12h/12h respectively). The water and bedding was autoclaved.

On the day of unloading, all mice were weighed. Subsequently, the tail rings of the NS and SUS mice were connected to a series of fish swivels with snap hooks, onto an eye bolt fastened to a slotted steel bar suspended atop a standard rat cage. The tail of the mouse was wrapped and taped onto the fish swivel. The snap swivel allowed for a 360 degree range of motion in the plane of the cage bottom. By adjusting the height of the eye bolt, the mice were free to move at the bottom of the cage at approximately a 30° angle (SUS mice) such that only the tips of the toes were in contact with the cage bottom, and as such, the hindlimbs were unloaded. The NS mouse tail rings were connected to the swivels but the mice were able to completely load on all four limbs. The NS and SUS mice were housed two mice per rat cage, one at either end, so they could not climb on each other. The NH mice were housed singly in a mouse cage, so they had the same volume of space and were housed alone similar to the NS and SUS mice without physical contact with other mice. (Fig 1). The bedding and cotton ‘nestlets’ were provided but reduced 1/3 to 1/2 in all groups in order to prevent the mice from climbing and loading their hindlimbs. The duration of the unloading experiment was 4 weeks.

At the end of the unloading period, mice were anesthetized with ketamine/dex-dormitor (75mg/kg and 0.5mg/kg respectively) and an intra-cardiac puncture for blood collection performed. The mice were then sacrificed by a rapid cervical dislocation. Their hindlimbs were excised and cleaned of muscle and connective tissues for further analysis.

### Corticosterone assay

All animals were sacrificed within a 4h window in the morning (11am ± 2 h). Blood obtained at sacrifice was allowed to clot and subsequently centrifuged 5 min at 3000g. The serum was aliquoted in new Eppendorf tubes and placed in -80°C until analysis. An ELISA corticosterone kit was purchased from DRG (EIA 4164, DRG International Inc, USA). The range of this kit is 0 to 240nmol/L and the sensitivity of this test is <1.63nmol/L. Intra and inter assay reproducibility are respectively 3% and 6%.

### Trabecular and cortical bone microarchitecture and microcomputed tomography analysis

Right tibiae were scanned *in vivo* at baseline, before the tail surgery and again 5 weeks later, at the end of the experiment *ex vivo*. After the sacrifice, the right tibiae were dissected and fixed in 4% paraformaldehyde for 1 day at +4°C. The next day, they were placed in 70% ethanol in a 15mL tube in packing material in order to prevent any movement. The specimens were scanned and analyzed using X-ray microCT (vivaCT40, Scanco Medical AG, Bassersdorf, Switwerland). Specimens were scanned at 55 kV, 145 μA, at the highest resolution scan parameter settings of 200 ms integration time, 1000 projections of 2048 samples within a 21.5 mm diameter field of view for the ex-vivo scans and 38.9mm diameter field of view for in-vivo scanning. The pixel size was 10.5 μm for the *ex vivo* scan and 19 μm for the *in vivo* scan (realized at baseline, before the tail ring surgery).

**Fig 2:**
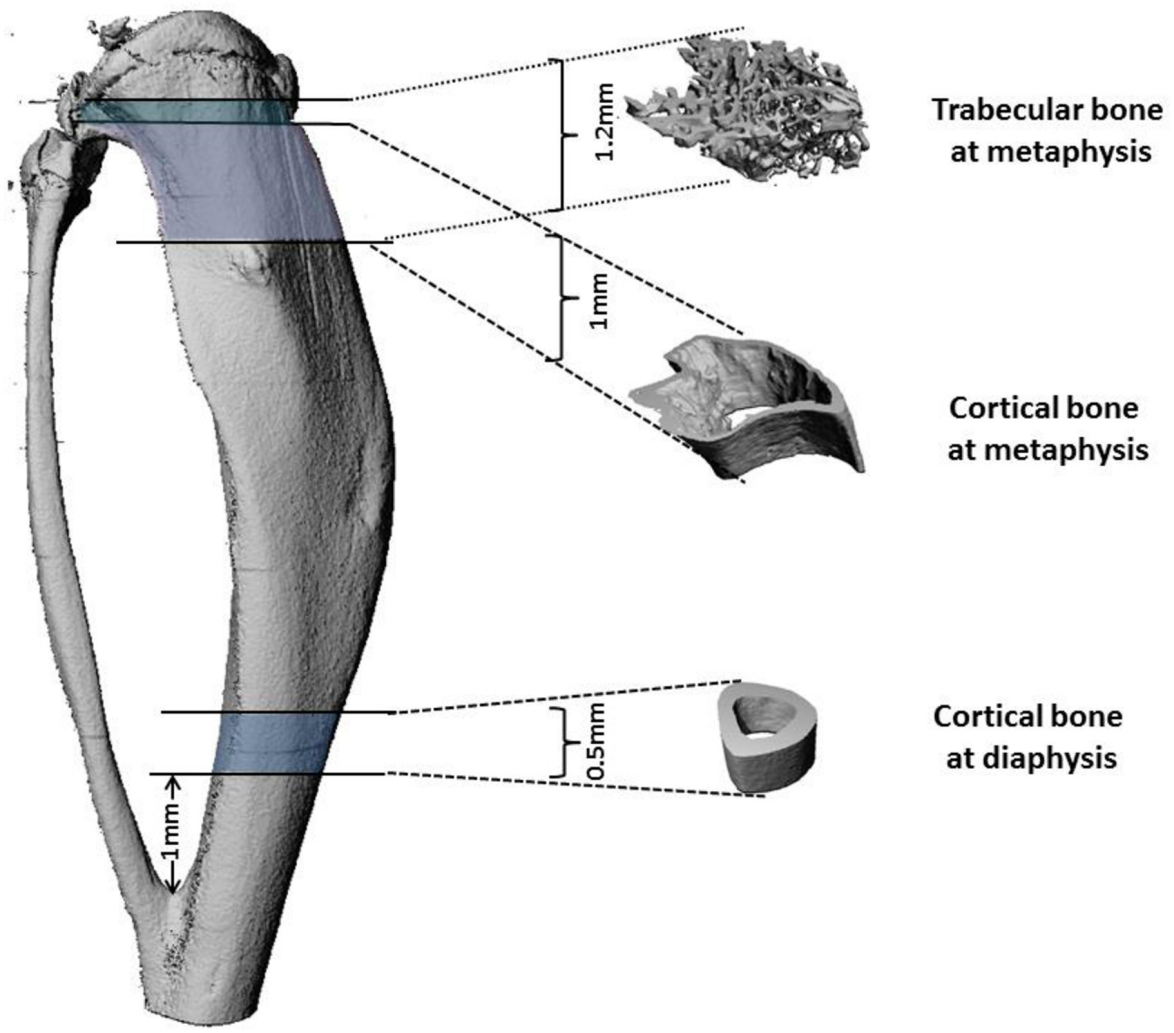
Trabecular and cortical bone regions analyzed by microCT. Trabecular bone microarchitecture was analyzed below the growth plate. 1.2mm was analyzed, which represents 63 slices *in vivo* (19 μm) and 120 slices *ex vivo* (10 μm voxel size). Cortical bone microarchitecture was analyzed below the growth plate (1mm analyzed representing 53 slices *in vivo* and 100 slices *ex vivo*) and above the tibia-fibula junction (0.5mm analyzed representing 26 sections *in vivo* and 50 sections -*ex vivo*).

For trabecular bone, three dimensional images within the range of 0 to 1.2 mm from the most proximal tibial metaphysis were reconstructed (Fig 2) using evaluation settings of threshold 270, Gauss Sigma 0.8 and Gauss Support 1.0. Bone morphometry was characterized by bone volume fraction value and density. Trabecular morphometry from the same region was further studied by excluding the cortical bone from the endocortical borders, and was characterized by bone volume fraction (BV/TV), density, trabecular thickness (Tb.Th), trabecular number (Tb.N) and trabecular spacing (Tb.Sp).

For cortical bone analysis, the same scan of the right tibiae used for trabecular bone quantitation were used for reconstruction. Approximately 1 mm in length of cortical bone was reconstructed in the metaphysis 0.2mm below the growth plate and 0.5 mm of the diaphysis was reconstructed starting 1mm above the tibia/fibula junction (Fig 2) using evaluation settings of threshold 355, Gauss Sigma 0.8 and Gauss Support 1.0. The parameters analyzed were the cortical thickness (Ct.Th), the cortical density and the cortical volume (BV/TV).

All the scans were analyzed in a blinded fashion. The trabecular bone was separated from the cortical bone manually, a few voxels removed from the endocortical surface, as described by Bouxsein et al. [32].

### Osteoclast number

Left hindlimbs were fixed with 4% paraformaldehyde at +4°C for 1 day, and then demineralized in 10–14% EDTA for 1 week. The bones were subsequently washed with water/PBS and dehydrated in different ethanol baths for 4hr each. Bones were then paraffin embedded and paraffin sections (5 μm) were cut, dewaxed, rehydrated and stained for tartrate-resistant acid phosphatase activity using the standard naphtol AS-BI phosphate postcoupling method (protocol from the University of Rochester Medical Center, Center for Musculoskeletal Research) with some minor modification. The slides were incubated for 45 minutes at 37°C in 0.92% sodium acetate buffer, pH 5.0, containing 0.004% naphtol AS-BI phosphate and 2.3% L-(+) Tartaric acid. Then the sections were incubated in the same buffer containing 0.1% pararosaniline chloride and 0.08% sodium nitrite for 10 minutes, followed by washing in distilled water. The sections were counterstained with methyl green for 4–6 minutes, dehydrated and coverslipped with Permount.

TRAP-positive osteoclasts were quantified in a blinded fashion using Osteomeasure software (OsteoMetrics, Inc., Decatur, GA, USA), and a digitalized tablet (Drawing Board III, GTCO CalComp by Turning Technologies, Scottsdale, AZ, USA). Osteoclast number (N.Oc/B.Pm) and osteoclast surface (Oc.S/BS) were obtained (20x objective magnification). The region of interest analyzed was 1 mm in length, 200μm below the growth plate, and 100μm from the cortical bone, corresponding to the area analyzed by microCT on the contralateral tibia.

### Statistical analysis

One-way Analysis of Variance was used to assess the differences between the groups regarding their housing state or genotype (NS and SUS mice, cKO HET and controls) followed by pairwise post hoc tests. Independent sample *t* test was used to compare the effect of cKO HET deletion in the NH groups (cKO HET versus controls). Independent sample *t* test was also used to compare the normal housing and non-suspended housing on both control and cKO HET mice. Separate analysis was conducted for male and female mice. In the aforementioned analyses, if the correlations between weight changes with bone phenotype parameters were significant, covariance analysis will then be conducted to account for the effect of weight. Pearson correlations were also conducted to determine correlation between weight changes, corticosterone levels and bone phenotype parameters. When examining the normality assumptions and equal variance assumptions between groups, the data showed mild violation. However parametric analysis methods were chosen over the non-parametric approach for the following reasons: First, the measurement was made at the ratio level which assumed normality based on probability theory. Second, the covariate analyses that was conducted to account for the effect of weight, can only be done with parametric statistics. Third, ANOVA has been shown to be robust with mild violation of assumptions. A *p* value of 0.05 or less was considered as statistically significant. All analysis was performed using IBM SPSS Statistics 22 (SPSS Inc., Chicago, IL, USA).

## Results

### Body weight

**Fig 3:**
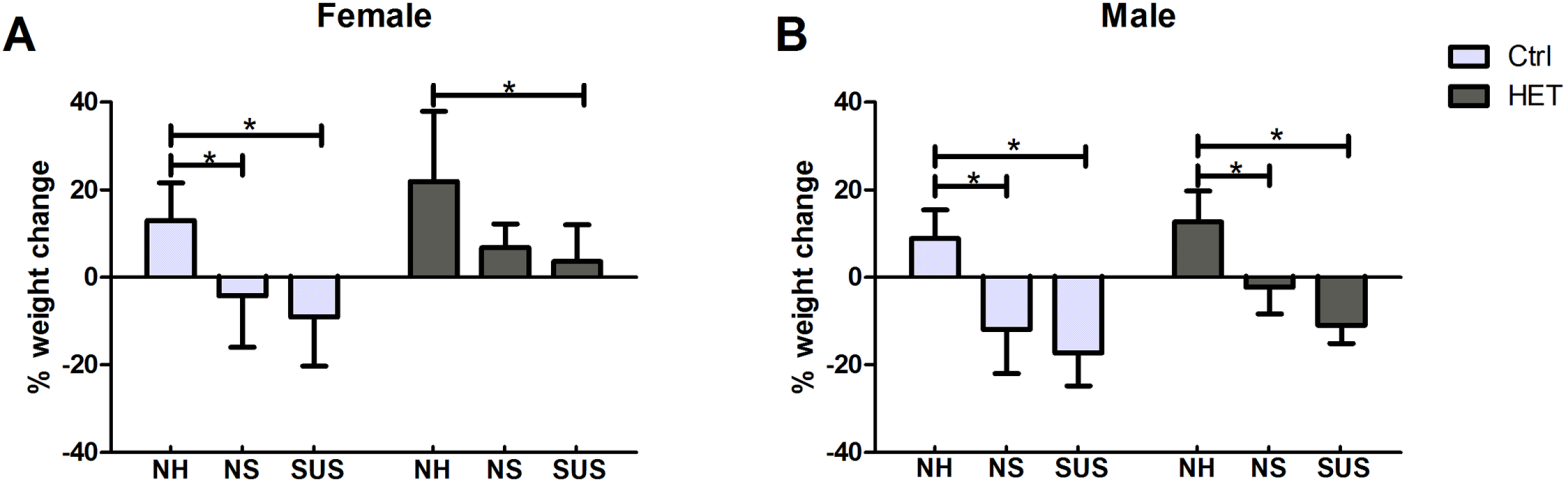
Body weight change for 3 different housing conditions and 2 genotypes. Ctrl: Control, HET: Dmp1 Cre x β-catenin heterozygotes, NH: Normal Housing, NS: Non Suspended, SUS: Suspended. Number of animals per group = 5–8. *: represents a significant difference between two groups (p<0.05). One-way Analysis of Variance was used to assess the differences between the groups followed by pairwise post hoc tests.

No significant differences in body weight were observed between males or females at 14 weeks of age before tail ring attachment. At the end of the experiment at 19 weeks, control and cKO HET male and female mice gained weight under normal housing conditions. However, under either non suspended conditions or suspended conditions, the control and cKO HET female (Fig 3A) and male (Fig 3B) mice did not gain weight or lost weight. Significant differences were observed between normally housed and non-suspended and suspended conditions. This shows that attachment and housing conditions, not unloading, had a significant effect on weight.

### Corticosterone

**Fig 4:**
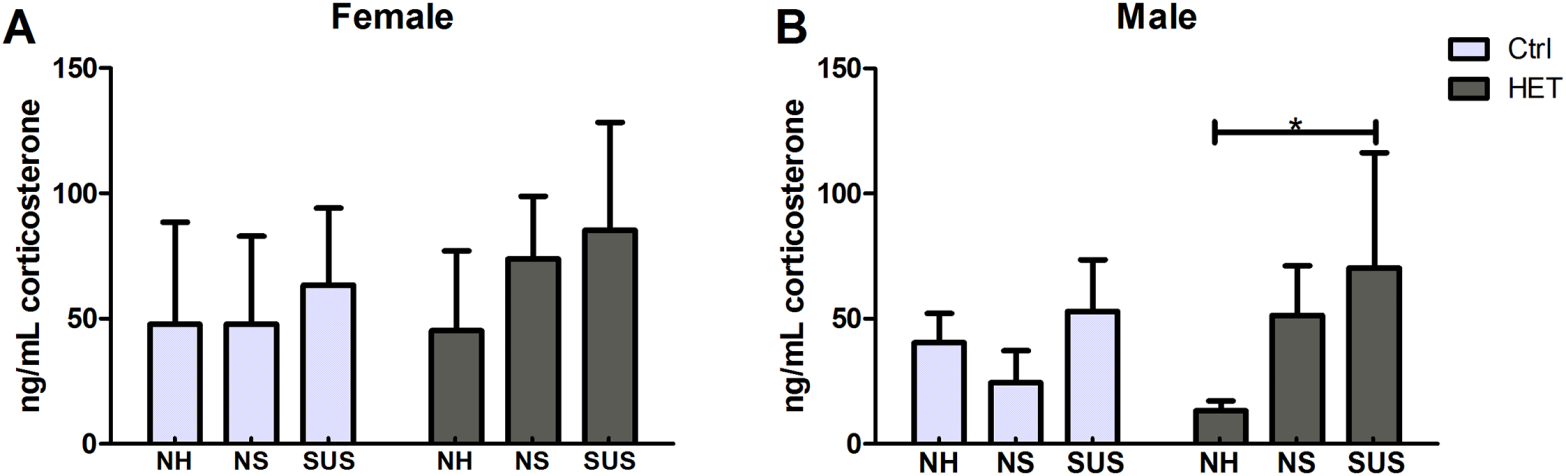
Corticosterone levels for 3 different housing conditions and 2 genotypes. Ctrl: Control, HET: Dmp1 Cre x β-catenin heterozygotes, NH: Normal Housing, NS: Non Suspended, SUS: Suspended. Number of animals per group = 5–8. *: represents a significant difference between two groups (p<0.05). One-way Analysis of Variance was used to assess the differences between the groups followed by pairwise post hoc tests.

Corticosterone is a steroid hormone produced by the adrenal glands in rodents, involved in the regulation of the stress response. To determine if a correlation existed between bone parameters and stress, corticosterone levels were measured. No differences were observed in the corticosterone levels of female control and cKO HET, or in male control mice. In male cKO HET mice, the suspended group had significantly higher levels of corticosterone compared to the normally housed group (NH/SUS: 13.31±3.85/70.29±46.00) (Fig 4A and 4B). No significant difference was observed between normal housing and attachment without suspension.

Correlations were performed between the bone parameters and corticosterone in males. No correlations were performed in females since there was no significant difference among the corticosterone levels. In males, the correlations revealed that corticosterone was inversely correlated with the cortical thickness at the diaphysis (p = 0.005; r = −0.45), the cortical thickness at the metaphysis (p = 0.01; r = -0.42), the trabecular BV/TV (p = 0.02; r = -0.39), the trabecular number (p = 0.03; r = -0.35), the trabecular thickness (p = 0.017; r = -0.39). Corticosterone levels were positively correlated with the trabecular spacing (p = 0.01; r = 0.41).

### Micro CT—Bone phenotype of the cKO HET male mice as compared to controls

#### Normal Housing, (NH)

A comparison of bone parameters were performed on 14 week old animals before experimentation and at 19 weeks at the end of the unloading experiments. A significant difference was observed in trabecular Tb.N (Ctrl/HET: 4.51±0.41/3.73±0.52mm) and Tb.Sp (Ctrl/HET: 0.23±0.02/0.29±0.05mm) for male cKO HET compared to controls at 14 weeks. No differences were observed in cortical bone (Table 1). At the end of the experiment, at 19 weeks of age, the same parameters were still significantly different Tb.N (Ctrl/HET: 4.88±0.51/3.95±0.44mm) and Tb.Sp (Ctrl/HET: 0.20±0.02/0.26±0.03mm), but significant differences were also observed in trabecular bone BV/TV (Ctrl/HET: 13.96±2.71/8.92±0.95) and Tb.Th. (Ctrl/HET: 0.05±0.00/0.04±0.00) and diaphyeal cortical thickness (Ctrl/HET: 0.21±0.01/0.19±0.01) (Table 1). Therefore the male cKO HET mice had less bone than the controls which decreases with age (between 14 and 19 wks). (Covariance analysis was not necessary as no significant correlation existed between weight and bone parameters).

**Table 1:** Bone phenotype of Control mice (Ctrl) as compared to β-catenin conditional knock-out heterozygous mice (cKO HET) at 14 weeks (*in vivo*) and 19 weeks (*ex vivo*).

Age	Position	Parameter	Female				Male			
			Ctrl		HET		Ctrl		HET	
			mean±SD	N	mean±SD	N	mean±SD	N	mean±SD	N
14 weeks	Diaphyseal Cortical	BMD (mg HA/ccm)	1060.11±13.90	6	1065.87±25.19	10	1058.99±12.79	14	1071.53±16.28	6
		BV/TV (%)	88.89±1.14	6	86.99±1.66*	10	90.08±1.74	14	89.78±2.56	6
		Ct.Th (mm)	0.21±0.01	6	0.20±0.01*	10	0.23±0.02	14	0.24±0.01	6
	Metaphyseal Cortical	BMD (mg HA/ccm)	925.98±22.37	8	933.6±29.77	17	925.5±14.69	14	938.56±20.45	13
		BV/TV (%)	75.44±2.77	8	75.18±6.09	17	76.32±3.70	14	78.68±2.57	13
		Ct.Th (mm)	0.12±0.01	8	0.12±0.01	17	0.12±0.01	14	0.13±0.01	13
	Metaphyseal Trabecular	BMD (mg HA/ccm)	753.36±17.82	9	757.29±27.13	17	730.53±18	14	720.05±25.64	14
		BV/TV (%)	8.71±1.47	9	8.12±2.10	17	19.42±4.53	14	19.93±1.76	14
		Tb.N (1/mm)	2.13±0.24	9	2.00±0.34	17	4.51±0.41	14	3.73±0.52*	14
		Tb.Th (mm)	0.06±0.00	9	0.06±0.01	17	0.07±0.01	14	0.07±0.00	14
		Tb.Sp (mm)	0.49±0.06	9	0.53±0.08	17	0.23±0.02	14	0.29±0.05*	14
19 weeks	Diaphyseal Cortical	BMD (mg HA/ccm)	1196.14±27.44	7	1169.43±24.24	6	1154.87±43.20	6	1149.55±32.64	6
		BV/TV (%)	91.32±0.51	7	90.47±0.34*	6	89.27±4.84	6	90.45±0.72	6
		Ct.Th (mm)	0.21±0.01	7	0.19±0.01*	6	0.21±0.01	6	0.19±0.01*	6
	Metaphyseal Cortical	BMD (mg HA/ccm)	1053.11±24.27	7	1047.59±42.81	6	1025.03±27.98	6	1013.88±27.52	6
		BV/TV (%)	85.40±1.62	7	84.54±1.68	6	81.97±1.82	6	80.48±7.39	6
		Ct.Th (mm)	0.13±0.01	7	0.12±0.01	6	0.10±0.01	6	0.10±0.01	6
	Metaphyseal Trabecular	BMD (mg HA/ccm)	913.24±30.76	7	916.49±54.78	6	877.35±15.46	6	877.87±25.64	6
		BV/TV (%)	8.95±1.29	7	7.71±2.34	6	13.96±2.71	6	8.92±0.95*	6
		Tb.N (1/mm)	2.65±0.29	7	2.54±0.53	6	4.88±0.51	6	3.95±0.44*	6
		Tb.Th (mm)	0.06±0.00	7	0.05±0.00	6	0.05±0.00	6	0.04±0.00*	6
		Tb.Sp (mm)	0.39±0.05	7	0.41±0.08	6	0.20±0.02	6	0.26±0.03*	6

Cortical bone mineral density (BMD), bone volume (BV/TV) and cortical thickness (Ct.Th) were analyzed at the diaphysis and at the metaphysis as shown in the Fig 2. Trabecular bone mineral density (BMD), bone volume (BV/TV), number (Tb.N), thickness (Tb.Th) and spacing (Tb.Sp) were analyzed at the metaphysis. N = number of animals per group.

*: represents a significant difference between Control and HET mice (p<0.05). Independent sample *t* test was used to compare the effect of HET deletion in the NH groups (HET versus controls). Data represents mean ± SD.

#### Effects of attachment and housing, (NS)

**Fig 5:**
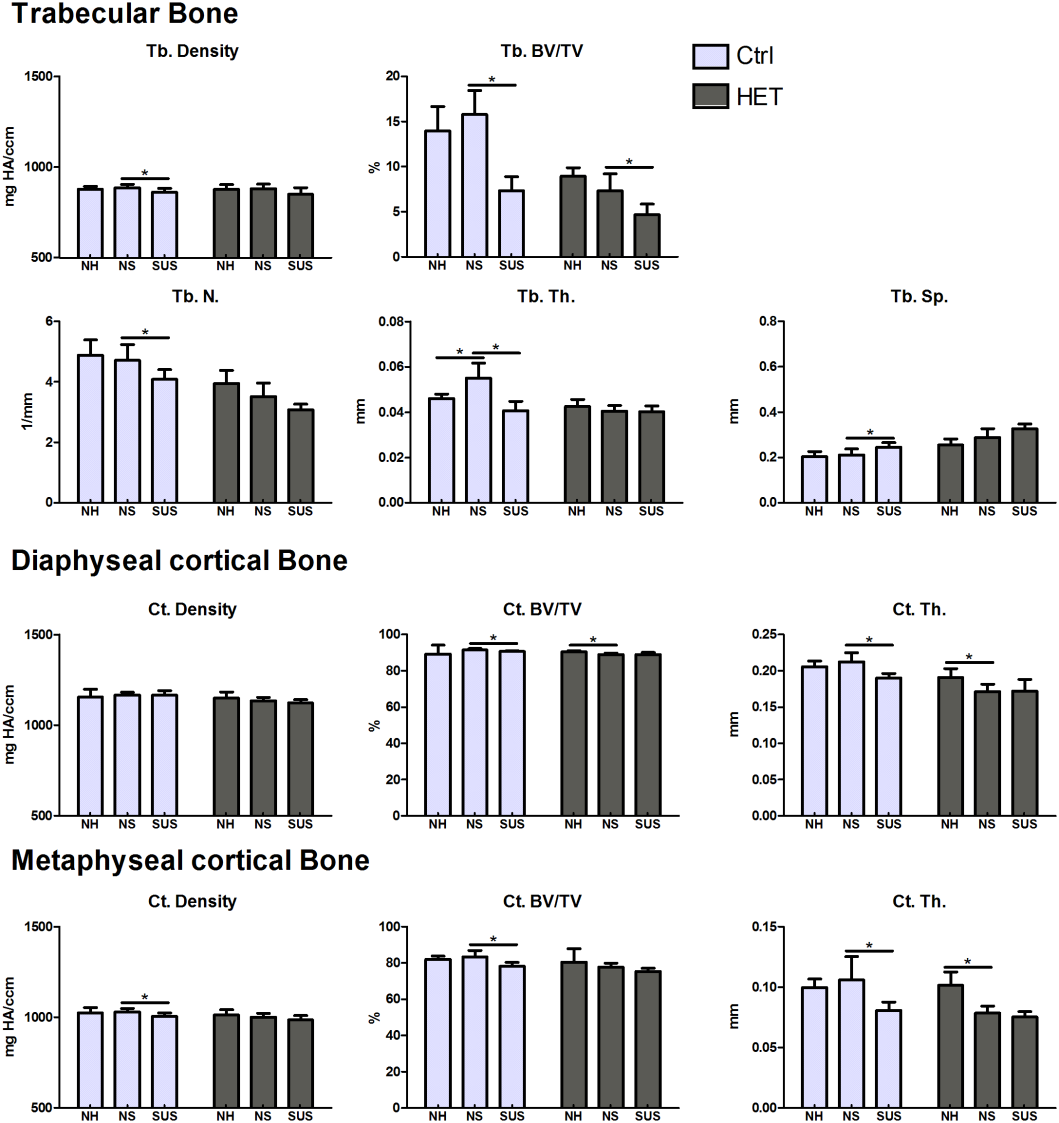
Effects of environment and suspension on 19 week old control and cKO HET male mice. Cortical bone mineral density (BMD), bone volume (BV/TV) and cortical thickness (Ct.Th) were analyzed at the diaphysis and at the metaphysis as shown in the Fig 2. Trabecular bone mineral density (BMD), bone volume (BV/TV), number (Tb.N), thickness (Tb.Th) and spacing (Tb.Sp) were analyzed at the metaphysis. *significant difference between NH and NS and between NS and SUS (p<0.05). One-way Analysis of Variance was used to assess the differences between the groups followed by pairwise post hoc tests. Number of animals per group = 5–7.

The bone phenotype of 19 week old animals housed under normal conditions (NH) was compared to animals attached, but not suspended (NS). In cKO HET males, diaphyseal cortical bone volume (NH/NS: 90.45±0.72/89.12±0.56%) and both diaphyseal (NH/NS: 0.19±0.01/0.17±0.01mm) and metaphyseal (NH/NS: 0.10±0.01/0.08±0.01mm) thickness were lower in the NS animals compared to the NH. Therefore, the cKO HET male mice lost bone under the non-suspended, but attached conditions. In the control mice trabecular Tb.Th was significantly higher in the NS compared to the NH (NS/NH:0.06±0.01/0.05±0.00) (Fig 5), however we didn’t observe significance after controlling for weight change in the analysis.

#### Effect of suspension/unloading (SUS)

In male control mice, as expected, cortical thickness (diaphysis and metaphysis) and volume (diaphysis and metaphysis), trabecular volume, density, number and thickness were significantly lower with suspension compared to non-suspended animals. In male cKO HET mice, the only significant difference observed was a moderate decrease in Tb BV/TV (NS/SUS: 0.07+ 0.02/ 0.05+0.01) (Fig 5).

### Micro CT—Bone parameters of the cKO HET female mice as compared to controls

#### Normal Housing (NH)

At 14 weeks of age, a significant difference was observed in diaphyseal cortical BV/TV (Ctrl/HET: 88.89±1.14/86.99±1.66%) and Ct. Th. (0.21±0.01/0.20±0.01mm) in the female mice as measured by *in vivo* microCT. This same difference was observed at 19 weeks of age for cortical BV/TV (Ctrl/HET: 91.32±0.51/90.47±0.34%) and Ct.Th. (0.21±0.01/0.19±0.01mm) as measured by *ex vivo* microCT. No significant differences were observed in metaphyseal cortical or trabecular bone at either 14 or 19 weeks of age (Table 1). Therefore, there was no significant decrease in bone with age.

#### Effects of attachment and housing, (NS)

**Fig 6:**
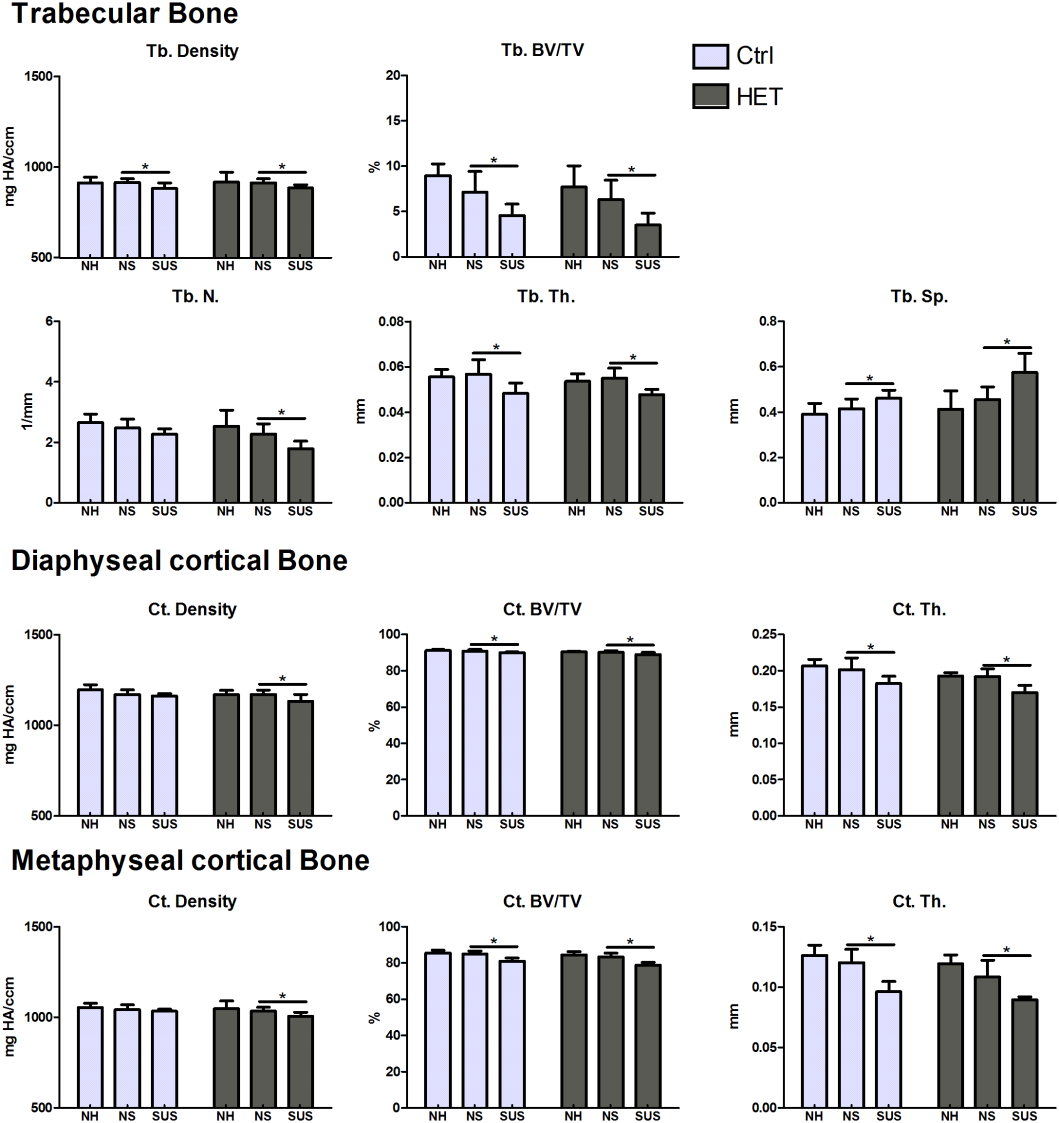
Effects of environment and suspension on 19 week old control and cKO HET female mice. Cortical bone mineral density (BMD), bone volume (BV/TV) and cortical thickness (Ct.Th) were analyzed at the diaphysis and at the metaphysis as shown in the Fig 2. Trabecular bone mineral density (BMD), bone volume (BV/TV), number (Tb.N), thickness (Tb.Th) and spacing (Tb.Sp) were analyzed at the metaphysis. *significant difference between NS and SUS (p<0.05). One-way Analysis of Variance was used to assess the differences between the groups followed by pairwise post hoc tests. Number of animals per group = 5–7.

The bone phenotype of 19 week old animals housed under normal conditions (NH) was compared to animals attached, but not suspended (NS). Unlike males, no differences were observed in females in any cortical and trabecular bone parameters between NH and NS groups, in either controls or cKO HET mice (Fig 6).

#### Effect of suspension/unloading (SUS)

In female control mice, cortical thickness (at both the diaphysis and metaphysis) and volume (diaphysis and metaphysis) values were significantly lower with suspension compared to non-suspended animals. The same parameters were also significantly decreased in the female cKO HET mice, in addition to a significant decrease in diaphyseal and metaphyseal cortical density, (diaphyseal: Ctrl/HET: 1170.43±25.74 to 1162.62±12.48/ 1169.84±24.41 to 1132.94±36.89 mg HA/ccm; metaphyseal Ctrl/HET:1142.70±25.61 to 1033.28±11.21/1034.02±21.11 to 1004.58±23.13 mg HA/ccm) with unloading (Fig 6). In female control mice, trabecular bone volume, density and trabecular thickness were lower with suspension compared to non-suspended controls, while the trabecular spacing was significantly higher. The same parameters were also significantly different between cKO HET females with suspension compared to non-suspended animals. In addition, trabecular bone number was significantly decreased (Ctrl/HET: 2.48±0.29 to 2.27±0.17 / 2.27±0.34 to 1.79±2.15 1/mm) (Fig 6). Therefore, based on diaplyseal and metaphyseal cortical density and trabecular number, the cKO HET females lost more bone than controls with suspension.

### Histological quantitation of osteoclast number and surface

Under non-suspended conditions, cKO HET males showed significant differences in osteoclast number compared to control male animals suggesting an effect of stress on cKO HET males. (Table 2).

**Table 2:** Osteoclast activity as measured by TRAP staining after sacrifice at 19 weeks.

Female	Parameter	NH		NS		SUS	
		Ctrl (n = 7)	HET(n = 6)	Ctrl (n = 8)	HET(n = 8)	Ctrl (n = 8)	HET(n = 8)
	N.Oc/B.Pm(1/mm)	8.7±2.0	8.2±3.3	7.6±1.8	8.2±2.3	7.8±1.8	8.3±3.7
	Oc.S/B.S(%)	21.1±5.1	18.8±6.5	16.9±4.8	19.8±5.5	15.8±4.4	18.5±5.9
Male	Parameter	NH		NS		SUS	
		Ctrl (n = 6)	HET(n = 6)	Ctrl (n = 7)	HET(n = 5)	Ctrl (n = 7)	HET(n = 6)
	N.Oc/B.Pm(1/mm)	6.6±0.9	7.1±3.2	4.8±1.3	8.1±1.9*	5.7±0.9	6.6±1.4
	Oc.S/B.S(%)	14.6±2.8	15.8±7.3	11.6±3.0	17.3±5.0	12.8±2.3	14.7±2.6

## Discussion

The goal of this study was to determine the role β-catenin plays in osteocytes under conditions of unloading of bone. Our hypothesis was that mice lacking one allele of β-catenin will lose more bone than the controls with unloading. This was found to be the case with females but not with males. Whereas greater bone loss was observed in the female cKO HETs compared to controls, the cKO HET males except for trabecular BV/TV. Gender differences were also observed in the bone phenotype of cKO HET males but not females compared to controls under normal housing conditions. Gender differences were also observed in cKO HET males, but not females in response to housing conditions/tail attachment. The lower bone mass due to phenotype and reduced bone due to stressful conditions in cKO HET male mice may preclude the loss of any additional bone due to unloading.

Deletion of one allele of β-catenin had a greater effect on tibial bone in males than in females. Under normal housing conditions male cKO HET mice have less cortical and trabecular bone than controls and females have less cortical but not trabecular bone compared to controls. Previous studies have reported a bone phenotype in female cKO HET mice and not in male cKO HET mice. In the paper by Kramer et al, a significant 5% reduction in trabecular bone was observed in female cKO HETs at 2 months of age [25]. In the Javaheri et al paper, a significant decrease was observed in both female and male cKO HETs at 18–24 weeks of age [26]. These differences could be due to the fact that the femur was analyzed in these two studies, whereas in the present study, the tibia were analyzed as this was the model used for unloading in our lab previously[33–34]. These observations suggest bone specific effects of allelic deletion of β-catenin.

Interestingly, cKO HET males were also more affected by the housing conditions than females. We observed lower cortical bone volume and thickness in non-suspended but attached cKO HET males compared to cKO HET males housed under normal conditions. By corticosterone analysis, the suspended cKO HET mice had significantly higher levels than the normally housed cKO HET mice suggesting greater stress for these transgenic animals under these conditions. No significant difference in corticosterone were found in control males or in control or cKO HET females. A recent unpublished study reported that normal wild-type males are more susceptible to stress than females [35]. It was reported than chronic mild stress induces bone loss through the glucocorticoid signaling pathway in osteoblasts and osteocytes in another transgenic mouse model [36]. Our studies suggest that β-catenin cKO HET male mice are even more susceptible to stress of housing conditions than wild-type animals.

Many of the papers that report effects of suspension on stress such as corticosterone levels or adrenal mass do not have the attached but not suspended control group, but instead have used normal housing as a control for suspended mice [37–39]. Based on the results from our study, attached but not suspended animals provides a complementary and useful control group to interpret results obtained from suspended animals. As expected, body weight in this study was lower in suspended mice compared to those housed normally. This effect of hindlimb unloading has been reported before [38,40]. However, we showed that the attached but not suspended animals also did not gain weight as compared to normally housed animals similar to the study by Gaignier et al [41]. Our studies suggest it is the attachment/housing that is responsible for lack of weight gain and not the suspension. Loss of body weight, including fat and muscle can lead to bone loss. In fact, it has been published that muscle atrophy following hindlimb unloading in mice happens before bone loss and may contribute to the skeletal deficits [38]. Interestingly, it has been shown that the temperature with animal facilities can have an effect on bone loss [42]. Mice housed for 2 months with no bedding at 32°C have higher trabecular bone volume compared to mice housed at 22°C. Since the attached/non-suspended and suspended mice have less bedding and cannot make physical contact, it is probable their thermoregulation is changed compared to mice housed in groups with normal bedding/housing. This is another reason for having the attached but non-suspended mice as controls.

With suspension, female controls lost both cortical bone volume and thickness and trabecular bone volume and thickness compared to attached/non-suspended mice. The same parameters were also lower in the cKO HET suspended mice in addition to lower cortical density and trabecular number with an increase in trabecular spacing. Therefore, the absence of one allele of β-catenin in females increases bone loss induced by suspension. This increase in bone loss may be due to an increase in RANKL and a decrease in OPG for the β-catenin cKO HOMO and HETs as described by Kramer and colleagues [25].

With suspension, cortical thickness, bone volume, and trabecular bone volume, number and thickness were significantly lower in control males compared to non-suspended control males. This is similar to results published previously by others [3,30]. Surprisingly, the cKO HET male mice only showed a minor but significant reduction in trabecular BV/TV with suspension as compared to non-suspended cKO HET mice. The cKO HET male mice had significantly less trabecular bone than controls under normal housing and significantly less cortical bone than controls exposed to attached/non-suspended conditions. This low bone mass was due to a phenotypic effect and potentially due to the stress of being housed singly with tail attachment. The amount of bone that was lost in controls with unloading may be equivalent to the amount of missing bone in the cKO HET male mice and the loss of bone due to stress. Therefore, it is not clear if β-catenin plays a role in bone loss due to unloading in males.

A gender difference in the β-catenin cKO HET animals has been reported previously in Kramer’s paper [25] (femur cancellous BV/TV), and in Javaheri’s paper[26](femur trabecular BV/TV, Tb.Sp). These differences may be due to hormonal interactions with the Wnt/β-catenin pathway. Such interactions between the Wnt/β-catenin pathway and the estrogen signaling pathway have been reported *in vitro* [43,44]and *in vivo* [34]. In mice, trabecular bone loss due to unloading is greater when ERα is deleted in osteocytes [34]. Moreover, trabecular bone density is lower in female ERα HET mice but not in males, suggesting ERα in females controls bone formation. In addition, crosstalk between Wnt/β-catenin and estrogen receptor signaling synergistically promotes osteogenic differentiation of mesenchymal progenitor cells [43]. It is not known if testosterone interacts with the Wnt/β-catenin pathway.

In conclusion, the deletion of one allele of β-catenin in osteocytes leads to greater bone loss in female mice following hindlimb unloading compared to controls. The cKO HET male mice appear to be more sensitive to stressful conditions, which resulted in lower cortical and trabecular bone compared to their control littermates. No significant additional bone loss occurred with unloading. This study also emphasizes the importance of controlling for stress induced by tail attachment during hindlimb unloading, especially for transgenic mice.
